# Longitudinal Course and Baseline Predictors of Trajectories of Clinician-assessed Adherence to Immunosuppressant Medication in Patients after Kidney Transplantation: A KTx360° Substudy

**DOI:** 10.1097/TXD.0000000000001813

**Published:** 2025-07-24

**Authors:** Martina de Zwaan, Mariel Nöhre, Felix Klewitz, Maximilian Bauer-Hohmann, Charlotte Kuczyk, Yesim Erim, Lena Schiffer, Deborah Meier, Julia K. Wolff, Uwe Tegtbur, Lars Pape, Mario Schiffer

**Affiliations:** 1 Department of Psychosomatic Medicine and Psychotherapy, Hannover Medical School, Hannover, Germany.; 2 Department of Psychosomatic Medicine and Psychotherapy, University Hospital Erlangen, Erlangen, Germany.; 3 Department of Nephrology and Hypertension, Hannover Medical School, Germany; 4 IGES Institute, Berlin, Germany.; 5 Department of Prevention Research and Social Medicine, Institute for Community Medicine, University Medicine Greifswald, Greifswald, Germany.; 6 Department of Sports Medicine, Hannover Medical School, Hannover, Germany.; 7 Department of Pediatrics II, University Hospital of Essen, University of Duisburg-Essen, Essen, Germany.; 8 Department of Nephrology and Hypertension, University Hospital Erlangen, Erlangen, Germany.

## Abstract

**Background.:**

Optimal and maintained adherence to immunosuppressive medication is essential to kidney graft success.

**Methods.:**

We analyzed the longitudinal course of immunosuppressant adherence as measured with the Basel Assessment of Adherence to Immunosuppressive Medication Scale interview for up to 3-y duration of the Kidney Transplantation 360° study. Additionally, we examined putative baseline predictors of adherence trajectories. During the investigation period, patients participated in a multidisciplinary aftercare program consisting of case management, psychosocial and exercise assessments and interventions, including telemedicine support.

**Results.:**

The analysis sample with at least 1 valid information on the Basel Assessment of Adherence to Immunosuppressive Medication Scale consisted of 838 adult patients (41.3% women) aged 52.3 y (SD 13.5). Adherence to immunosuppressants improved significantly during the Kidney Transplantation 360° aftercare program; however, at each assessment point, 17%–25% of the patients still reported suboptimal adherence. Baseline predictors for a better improvement of adherence were younger age, male sex, and a longer duration since transplantation. Those variables were associated with a lower adherence at baseline, and we detected a “catch-up effect” over time, which might have been supported by the comprehensive aftercare program.

**Conclusions.:**

We believe that our aftercare program has supported the “catch-up effect” in adherence in younger male patients with a longer time after transplantation. However, the lack of a control group limits causal interpretations.

## Graphical abstract

**Figure:**
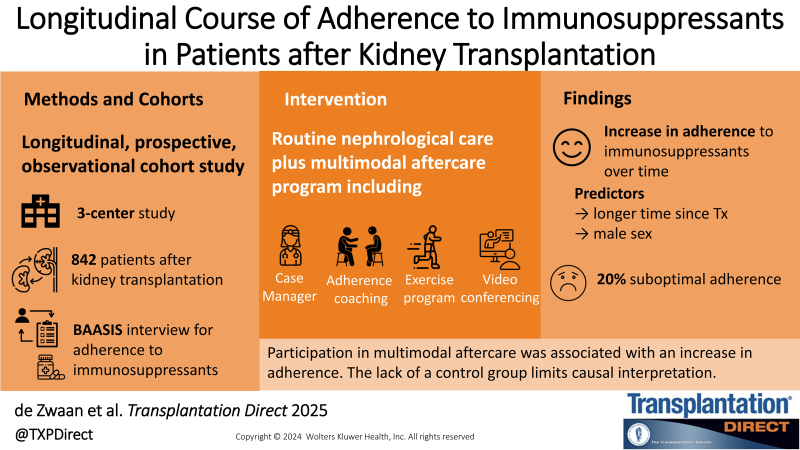

Solid organ transplantation is regarded as the best treatment option after irreversible organ failure. Taking the immunosuppressant medication, which is composed of the daily administration of 3 immunosuppressive drugs in the majority of cases at the correct time intervals and at the correct dose, is a critical part of posttransplant care. However, suboptimal adherence to immunosuppressant medication is frequent and can be a common reason for transplant failure.^1^ The importance of adhering to immunosuppressive therapy cannot be overemphasized, with the odds of organ failure increasing 7-fold for nonadherent recipients versus adherent recipients.^2^ Poor adherence to the immunosuppressant regimen is particularly high in renal transplant recipients compared with other organ transplant recipients (35.6 cases per 100 person-years versus 7 to 15 cases in other recipients).^3^

In cross-sectional studies, adherence is associated with several clinical characteristics although there are discrepancies between studies.^4^ Fairly consistently across studies, suboptimal adherence to the immunosuppressive regimen has been shown to increase over time, starting as early as 6 mo after transplantation,^5^ and to be higher in younger patients and male sex. Some but not all studies found an association between lower adherence and higher levels of depression and anxiety and lower levels of perceived social support. Other putative risk factors of nonadherence in renal transplant recipients have been summarized.^6-8^ Kidney Disease: Improving Global Outcome (KDIGO)^9^ categorized risk factors for nonadherence into 4 interrelated areas: patient/environment, caregiver, disease, and medication.

Adherence can be influenced by treatment. KDIGO^9^ recommends a team approach consisting of education, monitoring, recognition, and intervention. Shi et al^10^ could demonstrate in their meta-analysis of treatment studies that overall adherence can be improved in renal transplant recipients (relative risk, 1.23; 95% confidence interval, 1.08-1.41) and that multidisciplinary interventions showed higher efficacy compared with interventions by only 1 professional group (relative risk, 1.45; 95% confidence interval, 1.25-1.67). However, a recent Cochrane Review concluded that current evidence in support of interventions to increase adherence to immunosuppressant therapy is overall of low methodological quality, which is attributable to small sample sizes and heterogeneity for the types of interventions.^11^

Not much is known about the trajectories of adherence over time and about sociodemographic and clinical variables that predict adherence trajectories. Because medication adherence is a dynamic process, variables influencing its course are of importance for clinical care.

The goal of this investigation was to analyze the longitudinal course of medication adherence as measured with the Basel Assessment of Adherence to Immunosuppressive Medication Scale (BAASIS) interview over up to 3 y of participation in the Kidney Transplantation 360° (KTx360°) aftercare study.^12,13^ Additionally, we examined the association of sociodemographic and baseline psychosocial and medical indicators with BAASIS scores over time.

## PATIENTS AND METHODS

### Participants

Participants were adult patients after renal transplantation who participated in routine nephrological aftercare in the study centers at Hannover, Erlangen, and Hannoversch Münden in Germany. Incident patients had undergone KTx <1 y ago and were asked to participate directly after transplantation. Prevalent patients had received a transplant >1 y ago. Participation was offered to all patients regardless of sex, age, ethnicity, or socioeconomic status. Participants had to have statutory health insurance. Ethics approval was obtained from Hannover Medical School (number 3464-2017); the approval was confirmed by the ethics committees of all participating centers. Written informed consent was obtained from all participants before study entry.

### Intervention

KTx360° was a longitudinal, prospective, observational study. Details about the KTx360° aftercare program are described in previous publications.^12,13^ In short, in addition to routine nephrological care for patients after KTx, we offered a multimodal aftercare program consisting of adherence coaching, an individualized exercise program, and case management. As an important part of KTx360°, adherence assessments were regularly conducted using an interview format, and if positive for suboptimal adherence, adherence coaching was offered involving educational sessions by transplant nurses and/or individualized behavioral and psychosocial approaches conducted by psychologist or physician mental health experts. We applied blended care, which allowed us to use video conferencing with the patients. Behavioral and psychosocial interventions were limited to 8 sessions per year. Our goal was to implement patient-specific tailored interventions in a real-world setting, which usually has fewer resources than are available in clinical trials.^14,15^

### Instruments

#### Basel Assessment of Adherence to Immunosuppressive Medications Scale

The BAASIS is available as a self-report and an interview version (to be executed in a nonthreatening and nonjudgmental manner).^16^ We used the interview version of the 5-item BAASIS, which assesses adherence behavior regarding the intake of the immunosuppressants during the past 4 wk. The 3 items, “dose taking,” “drug holidays,” and “timing deviation from prescribed time,” are measured on a 6-point scale, which ranges from 0 (never) to 5 (every day). The 2 items, “change of dosing” and “stopping immunosuppressant medication intake,” are rated binary (1 = yes and 0 = no). We computed a total score for the 5 items, resulting in a continuous adherence rating ranging from 0 to 17 based on the evaluation used in other works^17,18^ and taking into account the current version of the BAASIS. In addition, nonadherence was dichotomously defined as any reported nonadherence on any of the 5 items (score >0).

#### Hospital Anxiety and Depression Scale

The German version of the Hospital Anxiety and Depression Scale (HADS), a self-report instrument, which is specifically designed to evaluate levels of anxiety and depression in physically ill patients, was used to measure levels of anxiety and depression at enrollment.^19-21^ The questionnaire consists of 2 subscales, “depression” and “anxiety,” with 7 items each. The items are rated between 0 and 3, leading to a sum score between 0 and 21, with higher results indicating higher levels of depression or anxiety. At baseline, Cronbach’s α in our sample was 0.857 for depression and 0.795 for anxiety.

#### Perceived Social Support Scale (F-SozU K7)

Perceived social support was measured using the German F-SozU K7 scale at enrollment.^22,23^ The instrument consists of 7 items rated on a 5-point Likert scale, ranging from 1 (does not apply) to 5 (exactly applicable). Therefore, a total score between 7 and 35 can be reached, with higher scores indicating higher perceived social support. At baseline, Cronbach’s α in our sample was 0.899.

#### Medical, Sociodemographic, and Transplant-specific Variables

The estimated glomerular filtration rate (eGFR) was extracted from the medical records at the time of enrollment into the study. In KTx recipients, the eGFR is an essential variable to define transplant functioning and to recognize difficulties regarding the transplanted organ, for example, organ rejection or overimmunosuppression. Sociodemographic and donation-specific variables, including sex, age at enrollment, partnership status, years of education, employment status, first language, donation type (living/deceased donor), previous KTx, and time since KTx at enrollment, were acquired using a self-report questionnaire. Missing information was taken from the medical records.

### Data Analysis

The BAASIS was analyzed in KTx360° participants using a longitudinal mixed model regression analysis with a log-transformed BAASIS score as the dependent variable (individuals on level 2 and their measurement points on level 1). Log transformation was conducted because of positively skewed data in the adherence measure. The change in BAASIS score over time (ie, potential intervention effect) was estimated by the fixed effect of the time in KTx360°. Level 2 covariates were sex, age at enrollment, years of education, employment status, partnership status, first language, donor type, previous KTx, time since KTx at enrollment, eGFR, and level of depression and anxiety (HADS) and perceived social support (F-SozU K7) at baseline. The mixed models include a random intercept, allowing for variation in intercept between individuals.

To examine the association of the above-mentioned level 2 covariates and BAASIS scores over time, we conducted separate analyses predicting BAASIS scores by the interaction of time in KTx360° and each covariate. Accordingly, we performed 13 mixed models, each with an interaction term for the respective covariate and time in KTx360°. To account for multiple comparisons, we applied the Bonferroni correction, adjusting the significance level to a *P* value of <0.004.

Furthermore, for covariates that revealed a significant association with the BAASIS score over time, we analyzed for incident and prevalent patients separately by either a 3-fold interaction in the regression model (for the predictor variable sex) or by a separate regression analysis only for the prevalent patients (for the predictor variable time since transplantation).

## RESULTS

### Participants

Study participants were recruited within the structured posttransplant care program KTx360° between May 2017 and July 2019.^12^ The project lasted until September 30, 2020, and took place in the transplant centers of Hannover Medical School, the hospital Hann. Münden in Lower Saxony and at the University Hospital in Erlangen, Germany. For the present analysis, we included adult participants, resulting in a sample of 937 participants. Eight hundred forty-two participants completed the BAASIS interview at least once (N = 838 were interviewed at baseline), with an average of 3.0 BAASIS assessments during the study (SD = 1.6, min = 1, max = 8). Because of missing data on the 13 covariates, the sample in the regression analyses was reduced to 683, with 199 (29.1%) incident (<1 y after transplantation at study enrollment) and 484 (70.9%) prevalent (>1 y after transplantation at study enrollment) participants. Descriptives of the total sample (n = 937), the sample with valid information on the BAASIS at baseline (n = 838), and the sample with complete data on all covariates (n = 683) are depicted in Table 1 and **Table S1** (**SDC,** https://links.lww.com/TXD/A767). Baseline characteristics are very similar in all samples, indicating no selection bias due to missing BAASIS assessments or missing covariates.

**TABLE 1.** Baseline characteristics of the entire sample, the reduced sample with missing data on covariates included in the regression analyses, and the incident and prevalent samples separately

	Sample with BAASIS score at baseline (N = 838)	Sample with complete data for regression analyses N = 683	Incident patients (N = 199)	Prevalent patients (N = 484)
Sex				
Female	346 (41.3%)	276 (40.4%)	80 (40.2%)	196 (40.5%)
Male	492 (58.7%)	407 (59.6%)	119 (59.8%)	288 (59.5%)
Age at enrollment, y				
Mean (SD)	52.3 (13.5)	52.2 (13.6)	49.9 (13.5)	53.1 (13.5)
Median (min–max)	54.0 (18.0–81.0)	54.0 (18.0–81.0)	51.0 (20.0–80.0)	55.0 (18.0–81.0)
Center				
MHH	607 (72.4%)	531 (77.7%)	173 (86.9%)	358 (74.0%)
NZN	184 (22.0%)	151 (22.1%)	26 (13.1%)	125 (25.8%)
ERL	47 (5.6%)	1 (0.1%)	0 (0%)	1 (0.2%)
Time in study, mo				
Mean (SD)	27.2 (9.36)	28.2 (9.05)	24.7 (10.4)	29.6 (8.03)
Median (min–max)	30.0 (0–41.0)	30.0 (0–41.0)	27.0 (0–40.0)	30.0 (0–41.0)
Time since transplantation, y				
Mean (SD)	5.32 (5.53)	5.76 (5.83)	1.00 (0)	7.71 (5.91)
Median (min–max)	3.00 (1.00–34.0)	4.00 (1.00–34.0)	1.00 (1.00–1.00)	6.00 (1.00–34.0)
First language				
German	704 (89.8%)	614 (89.9%)	176 (88.4%)	438 (90.5%)
Other	80 (10.2%)	69 (10.1%)	23 (11.6%)	46 (9.5%)
Missing	54	0	0	0
Partnership				
Yes	552 (69.2%)	480 (70.3%)	139 (69.8%)	341 (70.5%)
No	246 (30.8%)	203 (29.7%)	60 (30.2%)	143 (29.5%)
Missing	40	0	0	0
Years of education				
Mean (SD)	12.3 (2.45)	12.4 (2.44)	12.5 (2.66)	12.3 (2.34)
Median (min–max)	12.0 (8.00–18.0)	12.0 (8.00–18.0)	12.0 (8.00–18.0)	12.0 (8.00–18.0)
Missing	41	0	0	0
Work situation				
Not working	374 (48.8%)	323 (47.3%)	88 (44.2%)	235 (48.6%)
Part-time	173 (22.6%)	163 (23.9%)	48 (24.1%)	115 (23.8%)
Full-time	219 (28.6%)	197 (28.8%)	63 (31.7%)	134 (27.7%)
Missing	72	0	0	0
Donor type				
Living donation	239 (28.6%)	199 (29.1%)	48 (24.1%)	151 (31.2%)
Postmortem	598 (71.4%)	484 (70.9%)	151 (75.9%)	333 (68.8%)
Missing	1	0	0	0
Previous transplantation				
No	711 (85.1%)	581 (85.1%)	168 (84.4%)	413 (85.3%)
Yes	124 (14.9%)	102 (14.9%)	31 (15.6%)	71 (14.7%)
Missing	3	0	0	0
eGFR				
Mean (SD)	45.7 (18.4)	45.7 (18.0)	46.3 (16.8)	45.4 (18.5)
Median (min–max)	43.2 (10.3–123)	43.1 (11.2–123)	44.6 (15.2–111)	42.7 (11.2–123)
Missing	15	0	0	–
Anxiety (HADS-A)				
Mean (SD)	5.07 (3.85)	5.19 (3.90)	4.37 (3.55)	5.52 (3.99)
Median (min–max)	4.00 (0–19.0)	4.00 (0–19.0)	4.00 (0–17.0)	5.00 (0–19.0)
Missing	31	0	0	0
Depression (HADS-D)				
Mean (SD)	4.26 (3.88)	4.33 (3.98)	3.53 (3.70)	4.66 (4.05)
Median (min–max)	3.00 (0–21.0)	3.00 (0–21.0)	2.00 (0–19.0)	4.00 (0–21.0)
Missing	31	0	0	0
Perceived social support (F-SozU K7)				
Mean (SD)	30.1 (6.06)	30.0 (6.03)	31.2 (5.37)	29.6 (6.22)
Median (min–max)	32.3 (7.00–35.0)	32.0 (7.00–35.0)	33.0 (8.00–35.0)	32.0 (7.00–35.0)
Missing	86	0	0	0

eGFR, estimated glomerular filtration rate; ERL, University Hospital Erlangen; F-SozU K7, perceived social support scale; HADS, Hospital Anxiety and Depression Scale; MHH, Hannover Medical School; NZN, Transplant Center Hann. Münden.

### Adherence Trajectories

**FIGURE 1.**
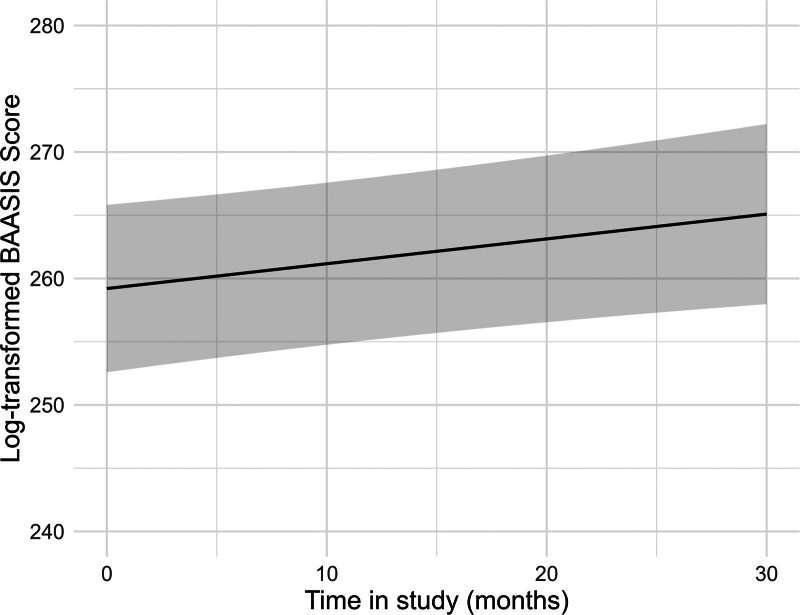
Estimated values of the log-transformed BAASIS total score over time, adjusted for all covariates. The regression analysis is depicted in Table 3. BAASIS, Basel Assessment of Adherence to Immunosuppressive Medication Scale.

Overall, adherence to immunosuppressant medication increased significantly over time (Figure 1 and Table 2). At each assessment point, between 17% and 25% of the participants did not show optimal adherence, with only small differences between taking and timing adherence (Table 3).

**TABLE 2.** Results of the multilevel regression analysis for log-transformed BAASIS total scores without interactions

Predictors	Estimates	95% CI	*P*
Intercept	259.55	252.97 to 266.14	**<0.001**
Time in KTx360°	0.20	0.04 to 0.35	**0.015** ^*a*^
Age at enrollment into the study	0.42	0.21 to 0.62	**<0.001**
Time since KTx at enrollment into study	–0.61	–1.01 to –0.21	**0.003**
Sex (male)	–5.07	–9.96 to –0.18	**0.042**
Work situation (part-time)	–0.38	–6.41 to 5.66	0.903
Work situation (full-time)	0.23	–5.92 to 6.38	0.941
Years of education	–1.62	–2.62 to –0.62	**0.002**
First language (non-German)	0.84	–6.80 to 8.49	0.829
Partnership (no)	–3.75	–9.28 to 1.77	0.183
Perceived social support (F-SozU K7)	0.30	–0.15 to 0.74	0.188
Anxiety (HADS-A)	–0.10	–0.94 to 0.74	0.813
Depression (HADS-D)	–0.56	–1.43 to 0.31	0.207
eGFR	0.07	–0.06 to 0.21	0.288
Donor type (postmortal)	0.14	–5.25 to 5.53	0.959
Previous KTx (yes)	6.62	0.11 to 13.12	**0.046**
Random effects			
σ^2^	1146.05		
τ_00_	500.03		
N	683		
Observations	2131		

Bold *P*-values indicate significant results (without Bonferroni correction).

Reference categories: sex = female, work situation = not working, first language = German, partnership = yes, donor type = living donation, and previous KTx = no.

^*a*^This indicates a significant change over time (the amount of time in the KTx360° study significantly predicted the BAASIS scores).

BAASIS, Basel Assessment of Adherence to Immunosuppressive Medication Scale; CI, confidence interval; eGFR, estimated glomerular filtration rate; F-SozU K7, perceived social support scale; HADS, Hospital Anxiety and Depression Scale; KTx, kidney transplantation.

**TABLE 3.** BAASIS sum score means (SD) and % of participants with optimal adherence (BAASIS = 0) at the different assessment points of the total sample of 842 with at least 1 BAASIS score

Time in study	Sample size (n)	BAASIS total score, mean	BAASIS total score, SD	% optimal adherence	% optimal taking adherence	% optimal timing adherence
Baseline	838	0.42	1.15	77.92	88.42	86.87
Month 3	137	0.23	0.58	83.21	91.24	90.51
Month 6	313	0.39	0.94	76.36	83.39	90.1
Month 9	171	0.35	0.91	78.95	92.4	85.38
Month 12	356	0.36	1.06	78.37	88.2	89.61
Month 18	284	0.34	0.93	77.82	87.68	88.73
Month 24	236	0.31	0.66	77.54	88.56	88.14
Month 30	118	0.34	1.10	82.20	93.22	88.14
Month 36	44	0.45	1.00	75.00	86.36	84.09

BAASIS, Basel Assessment of Adherence to Immunosuppressive Medication Scale.

**FIGURE 2.**
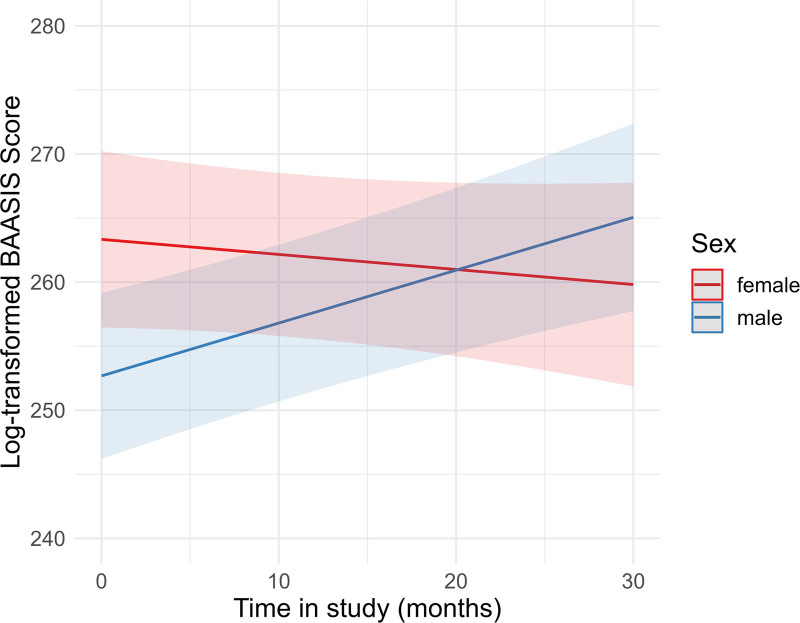
Estimated values of the interaction plot between BAASIS total score over time and sex, adjusted for all covariates. The regression analysis is depicted in Table 5. BAASIS, Basel Assessment of Adherence to Immunosuppressive Medication Scale.

**FIGURE 3.**
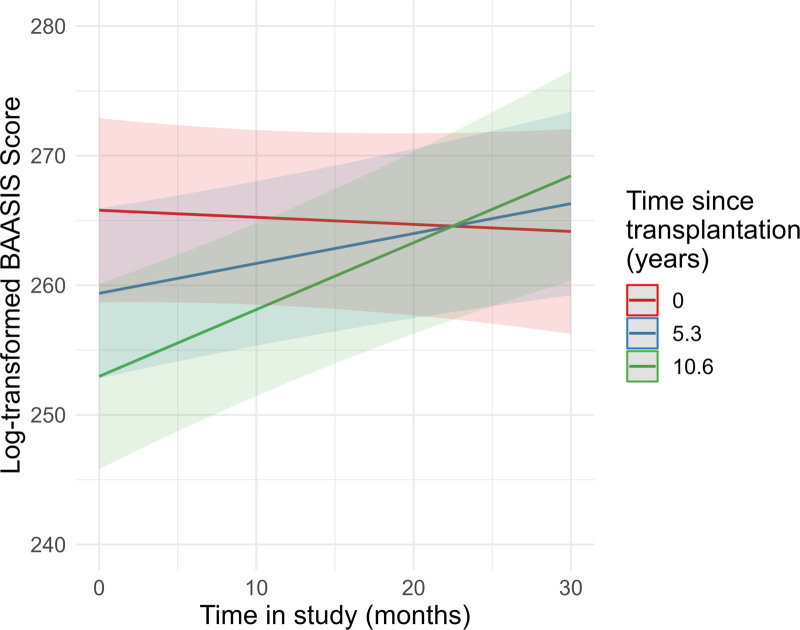
Estimated values of the Interaction plot between BAASIS total score over time and time since transplantation, adjusted for all covariates. The regression analysis is depicted in Table 6. BAASIS, Basel Assessment of Adherence to Immunosuppressive Medication Scale.

We performed multiple mixed model regression analyses to predict the log-transformed BAASIS total score by the covariates, that is, the interaction between time (months) in KTx360° and the respective predictor variable. After Bonferroni correction, 2 of 13 mixed models yielded significant interaction effects (Table 4). Results for sex revealed a significant interaction term (b = 0.53, *p* = 0.001), indicating that change in the BAASIS total score varies among men and women (Table 5 and Figure 2). With respect to time since transplantation, the interaction term was also significant (b = 0.05, *P* = 0.001; Table 6 and Figure 3). Results for age did not reach significance after Bonferroni correction (b = –0.01, *P* = 0.048; **Table S2 and Figure S1, SDC,** https://links.lww.com/TXD/A767). Results showed that male sex and longer time since transplantation were associated with increasing adherence over time.

**TABLE 4.** Regression results adjusted for all covariates on the interaction effects of time in study and each of the 13 covariates

	Estimates	95% CI	*P*
Age × time in study	–0.01	–0.02 to –0.00	**0.048**
Sex × time in study	0.53	0.21 to 0.85	**0.001**
Time since transplantation × time in study	0.05	0.02 to 0.08	**0.001**
Years of education × time in study	0.07	–0.00 to 0.13	0.053
eGFR × time in study	0.00	–0.01 to 0.01	0.482
Social support (F-SozU K7) × time in study	–0.01	–0.04 to 0.02	0.633
Anxiety (HADS-A) × time in study	0.00	–0.04 to 0.05	0.845
Depression (HADS-D) × time in study	–0.01	–0.05 to 0.04	0.773
Employment × time in study	0.21	–0.17 to 0.58	0.274
First language × time in study	–0.04	–0.56 to 0.47	0.870
Partnership × time in study	0.23	–0.11 to 0.57	0.177
Donor type × time in study	–0.27	–0.61 to 0.06	0.110
Previous transplantation × time in study	0.06	–0.37 to 0.49	0.786

Bold *P*-values indicate significant results (without Bonferroni correction).

Reference categories: sex = female, work situation = not working, first language = German, partnership = yes, donor type = living donation, previous KTx = no.

CI, confidence interval; eGFR = estimated glomerular filtration rate; F-SozU K7, perceived social support scale; HADS, Hospital Anxiety and Depression Scale.

**TABLE 5.** Sex predicts the course of the BAASIS score

Predictors	Estimates	95% CI	*P*
Intercept	262.63	255.70 to 269.55	**<0.001**
Time in KTx360°	–0.12	–0.36 to 0.13	0.351
Sex (male)	–10.66	–16.61 to –4.71	**<0.001**
Time since KTx at enrollment	–0.61	–1.01 to –0.21	**0.003**
Age at enrollment into study	–0.55	–6.59 to 5.49	0.858
Work situation (part-time)	0.14	–6.01 to 6.29	0.964
Work situation (full-time)	0.42	0.21 to 0.62	**<0.001**
Years of education	–1.65	–2.66 to –0.65	**0.001**
First language (non-German)	0.74	–6.91 to 8.38	0.850
Partnership (no)	–3.87	–9.40 to 1.65	0.169
Perceived social support (F-SozU K7)	0.31	–0.13 to 0.76	0.171
Anxiety (HADS-A)	–0.07	–0.91 to 0.78	0.878
Depression (HADS-D)	–0.57	–1.44 to 0.31	0.203
eGFR	0.07	–0.06 to 0.20	0.304
Donor type (postmortal)	0.12	–5.27 to 5.51	0.964
Previous KTx (yes)	6.62	0.12 to 13.13	**0.046**
Time in KTx360° × sex (male)	0.53	0.21 to 0.85	**0.001** ^*a*^
Random effects			
σ^2^	1139.68		
τ_00_	500.47		
N	683		
Observations	2131		

Bold *P*-values indicate significant results (without Bonferroni correction).

Multilevel regression analyses for log-transformed BAASIS total scores and change in BAASIS scores (depicted as the interaction between time in KTx360° and sex).

Reference categories: sex = female, work situation = not working, first language = German, partnership = yes, donor type = living donation, previous KTx = no.

^*a*^This indicates an interaction between change in BAASIS score over time and sex.

BAASIS, Basel Assessment of Adherence to Immunosuppressive Medication Scale; CI, confidence interval; eGFR, estimated glomerular filtration rate; F-SozU K7, perceived social support scale; HADS, Hospital Anxiety and Depression Scale; KTx, kidney transplantation.

**TABLE 6.** Time since transplantation predicts the course of the BAASIS score

Predictors	Estimates	95% CI	*p*
Intercept	258.97	252.37 to 265.57	**<0.001**
Time in KTx360°	0.22	0.06 to 0.37	**0.007** ^*a*^
Time since KTx at enrollment	–1.21	–1.74 to –0.68	**<0.001**
Sex (male)	–5.03	–9.91 to –0.14	**0.044**
Age at enrollment into study	–0.42	–6.45 to 5.60	0.890
Work situation (part-time)	0.28	–5.86 to 6.42	0.929
Work situation (full-time)	0.43	0.22 to 0.63	**<0.001**
Years of education	–1.66	–2.66 to –0.66	**0.001**
First language (non-German)	0.71	–6.92 to 8.34	0.855
Partnership (no)	–3.72	–9.23 to 1.80	0.186
Perceived social support (F-SozU K7)	0.30	–0.14 to 0.75	0.185
Anxiety (HADS-A)	–0.11	–0.95 to 0.74	0.805
Depression (HADS-D)	–0.55	–1.42 to 0.31	0.211
eGFR	0.07	–0.06 to 0.20	0.296
Donor type (postmortal)	0.11	–5.27 to 5.49	0.968
Previous KTx (yes)	6.62	0.13 to 13.11	**0.046**
Time in KTx360° × time since KTx	0.05	0.02 to 0.08	**0.001** ^*b*^
Random effects			
σ^2^	1140.44		
τ_00_	496.87		
N	683		
Observations	2131		

Bold *P*-values indicate significant results (without Bonferroni correction).

Multilevel regression analyses for log-transformed BAASIS total scores and change in BAASIS scores (depicted as the interaction between time in KTx360° and time since transplantation).

Reference categories: sex = female, work situation = not working, first language = German, partnership = yes, donor type = living donation, previous KTx = no.

^*a*^This indicates a significant change over time (the amount of time in the KTx360° study significantly predicted the BAASIS scores).

^*b*^This indicates an interaction between change in BAASIS score over time and time since transplantation.

BAASIS, Basel Assessment of Adherence to Immunosuppressive Medication Scale; CI, confidence interval; eGFR, estimated glomerular filtration rate; F-SozU K7, perceived social support scale; HADS, Hospital Anxiety and Depression Scale; KTx, Kidney Transplantation.

We did not find a differential effect between the incident and prevalent patients concerning the prediction of the course of the BAASIS score by sex (time in KTx360° × sex × group: b = –0.01, *P* = 0.986). With regard to time since transplantation, analyzing the prevalent patients separately revealed that the change in BAASIS total scores was comparable (b = 0.05, *P* = 0.012) to the total sample (see Table 7).

**TABLE 7.** Time since transplantation predicts the course of the BAASIS score, including only the prevalent patients

Predictors	Estimates	95% CI	*P*
Intercept	253.77	245.31 to 262.23	**<0.001**
Time in KTx360°	0.36	0.16 to 0.56	**<0.001** ^*a*^
Age at enrollment	0.63	0.36 to 0.89	**<0.001**
Time since KTx at enrollment	–0.87	–1.54 to –0.21	**0.010**
Sex (male)	–5.19	–11.65 to 1.27	0.115
Work situation (part-time)	–1.45	–9.35 to 6.45	0.719
Work situation (full-time)	–2.21	–10.47 to 6.05	0.599
Years of education	–2.14	–3.53 to –0.76	**0.002**
First language (non-German)	5.07	–5.14 to 15.28	0.329
Partnership (no)	0.10	–7.15 to 7.35	0.978
Perceived social support (F-SozU K7)	0.15	–0.39 to 0.70	0.588
Anxiety (HADS-A)	–0.05	–1.16 to 1.06	0.933
Depression (HADS-D)	–0.54	–1.68 to 0.60	0.350
eGFR	0.09	–0.08 to 0.26	0.288
Donor type (postmortal)	–0.03	–6.80 to 6.74	0.993
Previous KTx (yes)	8.45	–0.21 to 17.12	0.056
Time in KTx360° × time since KTx	0.05	0.01 to 0.08	**0.012** ^*b*^
Random effects			
σ^2^	1239.32		
τ_00_	638.50		
N	484		
Observations	1436		

Bold *P*-values indicate significant results (without Bonferroni correction).

Reference categories: sex = female, work situation = not working, first language = German, partnership = yes, donor type = living donation, previous KTx = no.

^*a*^This indicates a significant change over time (the amount of time in the KTx360° study significantly predicted the BAASIS scores).

^*b*^This indicates an interaction between change in BAASIS score over time and time since transplantation.

BAASIS, Basel Assessment of Adherence to Immunosuppressive Medication Scale; CI, confidence interval; eGFR, estimated glomerular filtration rate; F-SozU K7, perceived social support scale; HADS, Hospital Anxiety and Depression Scale; KTx, kidney transplantation.

As can be seen in the model without interactions (Table 2), the covariate “years of education” was negative and the variable “previous KTx” was positively associated with overall adherence but not with the trajectory of adherence over time (no interaction effect with “time in KTX360°”; Table 4). No associations were found with levels of depression, anxiety and perceived social support, partnership and work status, kidney function, and donor type.

## DISCUSSION

Adherence to immunosuppressive medication increased significantly over time in renal transplant patients participating in the multimodal aftercare program KTx360°. Because we could not include a control group due to funding agency regulations, we cannot definitively attribute this increase to the effect of the aftercare program itself. However, variables that were significantly and negatively associated with adherence at the time of enrollment into the intervention were positive predictors for an increase in adherence during the aftercare intervention. These variables, that is, younger age, male sex, and specifically longer time since transplantation, have repeatedly been shown to be associated with suboptimal adherence. Without a specific aftercare intervention, one would not expect an increase in adherence if the patients had just received nephrological treatment as usual. It is reasonable to assume that such a “catch-up effect” might have been supported by our program, either by simply monitoring adherence regularly (reactive effect), by the specific adherence interventions, or by the entire multimodal program. It has been described before that the observation itself might positively influence the outcome, in this case, adherence (Hawthorne effect).^24,25^

Sex, age, and time posttransplantation are nonmodifiable risk factors and cannot be addressed in interventions. However, they might point to patients with an increased risk for nonadherence. KDIGO^9^ recommends “to consider providing KTx patients at increased risk for nonadherence with increased levels of screening for nonadherence.”

Although the improvement of adherence was significant over time, roughly 20% did not show optimal adherence at any given time point over the KTx360° intervention period. Although this nonadherence rate is somewhat lower compared with the rates presented in the literature,^26^ this is not satisfactory. However, it is important to keep in mind that the BAASIS is a diagnostic instrument with a low threshold when it comes to identifying suboptimal adherence. A patient who missed 1 intake of a twice-daily dose during the last 4 wk might still have an individual taking adherence of 98% (55/56 doses = 98.2%) during this period. This strict standard is justified as there is evidence that even with a taking adherence rate of <98%, a worsening in clinical outcome can be expected.^27-29^ This suggests that a near-perfect adherence is essential for reduced patient morbidity.^11^ This points to the need for a more focused and intensive intervention for patients with demonstrated suboptimal adherence. However, our aftercare program was not solely focused on adherence but also on physical fitness, psychosocial well-being, and improved case management.

In contrast, KDIGO^9^ has recommended taking into account the threshold of the effect of nonadherence on the therapeutic outcome and defines nonadherence as “deviation from the prescribed medication regimen sufficient to adversely influence the regimen’s intended effect.” After KTx, the standard immunosuppressive regimen usually consists of tacrolimus, mycophenolate mofetil, and low-dose corticosteroids. Although these drugs are known to give the best outcome and survival rates, they are also known for their narrow therapeutic window and large inter‐patient variability, making it difficult to identify the right dose for each individual patient. Monitoring drug levels is common clinical practice but does not always reflect the pharmacological activity of these drugs accurately and might lead to under‐ or over‐immune suppression. To find the balance between optimal efficacy and minimal toxicity, it might be more informative to monitor patients’ immunological status rather than drug concentrations.^30^ Therefore, because the ideal individual dose of immunosuppressant medication is not really known (no biomarkers measuring immunocompetence are available), only studies prospectively investigating the relationship between nonadherence and outcomes will be able to unravel how much nonadherence is enough to result in deleterious clinical outcomes.^6^ Clearly, our study participants had been adherent enough to maintain a functioning allograft for a median of 3 y before study entry and in prevalent patients even for a median of 6 y (Table 1).

In agreement with earlier studies, we did not find an influence of donor type and renal function (eGFR) at baseline on adherence or adherence trajectories. Psychosocial aspects such as partnership, work situation, perceived social support, and levels of depression and anxiety were not associated with the overall adherence nor with the trajectory of adherence over time in our sample. Several cross-sectional studies have found an association between psychosocial variables and adherence using univariate analyses. Usually, the effect sizes of these associations are small^3^ and frequently disappear in adjusted, multivariate models.^31^ However, specifically, psychological variables can change over time and might exert an influence on adherence depending on their own course over time.

Addressing medication nonadherence in routine clinical practice is challenging; there is no method without limitations and no approach can be regarded as a gold standard.^32^ Strategies include self-report, collateral reports, pill counts, prescription refills, electronic medication monitoring devices, and biochemical measures (eg, immunosuppressant trough levels and their intrapatient variability).^7,9,33^ Currently, electronic medication monitoring devices are considered to be the best and most reliable method to assess adherence to medication in clinical studies, but they are cost-prohibitive for routine use.^34^ Additionally, we and others have found that self-reported adherence might be a good enough estimate of medication adherence.^3,35^ There is even evidence that studies in which adherence was measured using self-rating instruments showed the highest nonadherence rates.^3^ A number of validated questionnaires, such as the BAASIS, are available, either as self-report or third-party rating instrument.^32^ Ideally, >1 approach to measure adherence should be used.^9^

### Strengths and Limitations

We followed a large group of patients without the selection bias of randomized controlled studies over time with multiple assessment points using a well-validated and reliable instrument to assess adherence. Our intervention focused on patients with suboptimal adherence as there has been concern that studies targeting patients who are already adherent might lead to overimmunosuppression.^36^

We did not include a control group, which does not allow us to interpret the overall significant improvement in adherence ratings as a consequence of the intervention.

We do not know the long-term trajectories of medication adherence after the completion of our program, and, more importantly, the effect of adherence trajectories on clinical outcomes (eg, graft survival) remains unclear.

With regard to predictors for the course of the BAASIS, other potential risk factors for suboptimal adherence, such as health literacy, cognitive capacity, medication beliefs, or overall patient lifestyle, were not included in the regression analyses. The baseline values, as well as the course of these factors over time, might exert an influence on adherence trajectories.^37^

In conclusion, we believe that our aftercare program has supported the catch-up effect in adherence in younger male patients who have a longer time after transplantation. However, the lack of a control group may compound the accuracy of measuring the program effect. Efforts to promote kidney graft longevity have become a global interest, given the impact on quality of life, longevity, and costs. Adherence to immunosuppressive medication remains a relevant factor in the aftercare of patients with organ transplantation. Adherence is a dynamic process and may change over time. Therefore, it is necessary to continuously monitor adherence to whatever measures are used and that interventions be initiated or reinitiated.

## ACKNOWLEDGMENTS

The authors thank the other members of the KTx360° study group (Petra Anders, Johanna Boyen, Andrea Dehn-Hindenberg, Jan Falkenstern, Judith Kleemann, Dieter Haffner, Melanie Hartleib-Otto, Hermann Haller, Nils Hellrung, Nele K Kanzelmeyer, Christian Lerch, Marietta Lieb, Anna–Lena Mazhari, Martina Meiβmer, Regine Pfeiffer, Sandra Reber, Stefanie Schelper, Eva Kyaw Tha Tun, and Marit Wenzel) for their continuous support. Finally, they greatly appreciate the willingness of patients to participate in this study. Without them, KTx360° would not have been possible.

## Supplementary Material


