# Supplementary material for: Longitudinal Course and Baseline Predictors of Trajectories of Clinician-assessed Adherence to Immunosuppressant Medication in Patients after Kidney Transplantation: A KTx360° Substudy
Source: Transplant Direct. 2025 Jul 24;11(8):e1813. doi: 10.1097/TXD.0000000000001813 (PMC12289140; doi:10.1097/TXD.0000000000001813)

Supplemental Digital Content (SDC)

Table S1 Comparison between total adult KTx360° sample and, sample with a BAASIS assessment at baseline and sample with complete data included into the regression analyses

	**Entire adult KTx360° study sample N=937**	**Sample with BAASIS score at baseline N=838**	**Sample with complete data for regression analyses N=683**
Sex			
female	383 (40.9%)	346 (41.3%)	276 (40.4%)
male	553 (59.1%)	492 (58.7%)	407 (59.6%)
Missing	1	0	0
Age at enrollment, years			
Mean (SD)	52.6 (13.7)	52.3 (13.5)	52.2 (13.6)
Median [Min, Max]	55.0 [18.0, 81.0]	54.0 [18.0, 81.0]	54.0 [18.0, 81.0]
Center			
MHH	651 (69.5%)	607 (72.4%)	531 (77.7%)
NZN	232 (24.8%)	184 (22.0%)	151 (22.1%)
ERL	54 (5.8%)	47 (5.6%)	1 (0.1%)
Time in study, months			
Mean (SD)	26.0 (10.2)	27.2 (9.36)	28.2 (9.05)
Median [Min, Max]	29.0 [0, 41.0]	30.0 [0, 41.0]	30.0 [0, 41.0]
Time since transplantation, years			
Mean (SD)	5.33 (5.57)	5.32 (5.53)	5.76 (5.83)
Median [Min, Max]	3.00 [1.00, 34.0]	3.00 [1.00, 34.0]	4.00 [1.00, 34.0]
Donor type			
Living donation	262 (28.0%)	239 (28.6%)	199 (29.1%)
postmortem	673 (72.0%)	598 (71.4%)	484 (70.9%)
Missing	2	1	0
Previous transplantation			
no	791 (84.7%)	711 (85.1%)	581 (85.1%)
yes	143 (15.3%)	124 (14.9%)	102 (14.9%)
Missing	3	3	0
Graft failure	25	18	-

Table S2. Age at enrollment predicts the course of the BAASIS score. Multilevel regression analyses for log transformed BAASIS total scores and change in BAASIS scores (depicted as the interaction between time in KTx360° and age).

Predictors	***Estimates***	***CI***	***p***
Intercept	259.31	252.70 – 265.92	**<0.001**
Time in KTx360°	0.18	0.03 – 0.34	**0.023**§
Age at enrollment into study	0.54	0.30 – 0.78	**<0.001**
Time since KTx at enrollment into study	-0.62	-1.02 – -0.21	**0.003**
Sex [male]	-5.10	-10.00 – -0.21	**0.041**
Work situation [part-time]	-0.41	-6.45 – 5.63	0.894
Work situation [full-time]	0.35	-5.81 – 6.51	0.911
Years of education	-1.61	-2.61 – -0.61	**0.002**
First language (non-German)	1.03	-6.63 – 8.68	0.792
Partnership [no]	-3.86	-9.39 – 1.67	0.171
Perceived social support (F-SozU K7)	0.30	-0.14 – 0.75	0.184
Anxiety (HADS-A)	-0.08	-0.92 – 0.76	0.853
Depression (HADS-D)	-0.56	-1.43 – 0.31	0.207
eGFR	0.07	-0.06 – 0.20	0.299
Donor type [postmortal]	0.13	-5.26 – 5.53	0.961
Previous KTx [yes]	6.56	0.04 – 13.07	**0.048**
Time in KTx360° * Age at enrollment	-0.01	-0.02 – -0.00	**0.048**§§
**Random Effects**			
σ^2	1143.82		
τ_{00}	500.75		
N	683		
Observations	2131		

§This indicates a significant change over time (the amount of time in the KTx360° study significantly predicted the BAASIS scores).

§§This indicates an interaction between change over time and age

eGFR = estimated glomerular filtration rate. F-SozU K7 = perceived social support scale. HADS = Hospital Anxiety and Depression Scale

Reference categories: sex = female, work situation = not working, first language = German, partnership = yes, donor type = living donation, previous KTx = no

Figure S1. Estimated values of the Interaction plot between BAASIS total score over time and age at enrollment, adjusted for all covariates. The regression analysis is depicted in Supplemental Table 2.

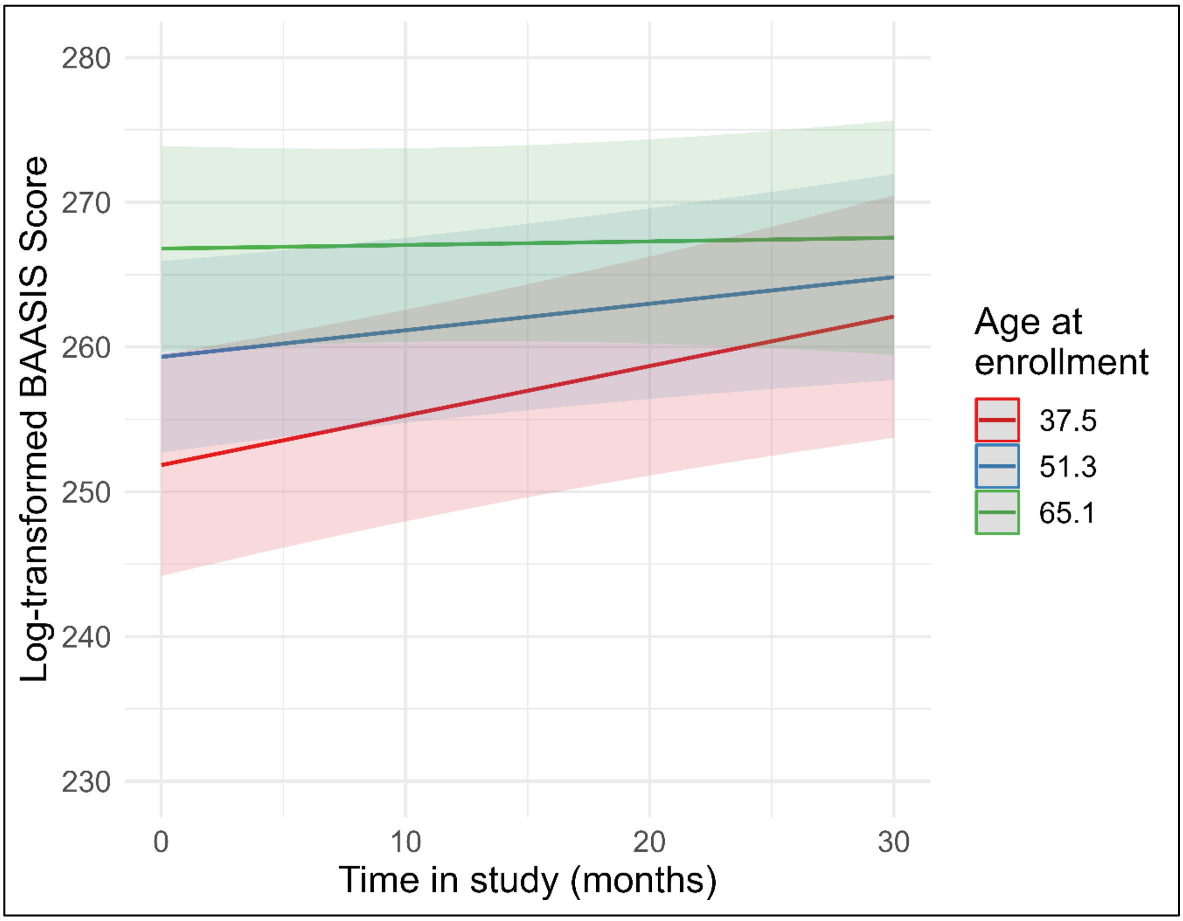

Supplement: Supplementary file 1 [file txd-11-e1813-s001.pdf]
